# Participation of MCP-induced protein 1 in lipopolysaccharide preconditioning-induced ischemic stroke tolerance by regulating the expression of proinflammatory cytokines

**DOI:** 10.1186/1742-2094-8-182

**Published:** 2011-12-24

**Authors:** Jian Liang, Jing Wang, Yasser Saad, Logan Warble, Edilu Becerra, Pappachan E Kolattukudy

**Affiliations:** 1 Burnett School of Biomedical Sciences, University of Central Florida College of Medicine, 4000 Central Florida Blvd. Orlando, FL 32816, USA

**Keywords:** Ischemic stroke, lipopolysaccharide (LPS) preconditioning, monocyte chemotactic protein-induced protein 1 (MCPIP1), middle cerebral artery occlusion (MCAO), proinflammatory cytokines

## Abstract

**Background:**

Lipopolysaccharide (LPS) preconditioning-induced neuroprotection is known to be related to suppression of the inflammatory response in the ischemic area. This study seeks to determine if **m**onocyte **c**hemotactic **p**rotein-**i**nduced **p**rotein 1 (MCPIP1), a recently identified CCCH Zn finger-containing protein, plays a role in focal brain ischemia and to elucidate the mechanisms of LPS-induced ischemic brain tolerance.

**Methods:**

Transcription and expression of MCPIP1 gene was monitored by qRT-PCR and Western blot. Mouse microglia was prepared from cortices of C57BL/6 mouse brain and primary human microglia was acquired from Clonexpress, Inc. Wild type and MCPIP1 knockout mice were treated with LPS (0.2 mg/kg) 24 hours before brain ischemia induced by transient middle cerebral artery occlusion (MCAO). The infarct was measured by 2,3,5-triphenyltetrazolium chloride (TTC) staining.

**Results:**

MCPIP1 protein and mRNA levels significantly increased in both mouse and human microglia and mouse brain undergoing LPS preconditioning. MCPIP1 mRNA level significantly increased in mice ipsilateral brain than that of contralateral side after MCAO. The mortality of MCPIP1 knockout mice was significantly higher than that of wild-type after MCAO. MCPIP1 deficiency caused significant increase in the infarct volume compared with wild type mice undergoing LPS preconditioning. MCPIP1 deficiency caused significant upregulation of proinflammatory cytokines in mouse brain. Furthermore, MCPIP1 deficiency increased c-Jun N terminal kinase (JNK) activation substantially. Inhibition of JNK signaling decreased the production of proinflammatory cytokines in MCPIP1 knock out mice after MCAO.

**Conclusions:**

Our data indicate that absence of MCPIP1 exacerbates ischemic brain damage by upregulation of proinflammatory cytokines and that MCPIP1 participates in LPS-induced ischemic stroke tolerance.

## Background

Stroke is the second leading cause of death and the most frequent cause of permanent disability worldwide [1]. Inflammatory mechanisms that are activated within hours after brain ischemia represent a key target of current translational ischemic stroke research [2]. It has been reported that the levels of proinflammatory cytokines and chemokines are increased after focal ischemia. Chemokines are cytokines that have the ability to induce chemotaxis on neighboring cells, particularly those involved in inflammatory actions [3,4]. While some cytokines may offer protection, many cytokines and most chemokines have been shown to participate in the neuronal damage processes [4,5]. Upregulation of cerebral proinflammatory cytokines, activation of local microglia, astrocytes and systemic lymphocytes and invasion of leukocyte in the brain contribute substantially to ischemic brain damage [6].

Published data have shown that lipopolysaccharide (LPS) preconditioning is a powerful neuroprotective phenomenon by which a sublethal injurious stimulus renders the brain resistant to a subsequent damaging ischemic insult [7-11]. LPS preconditioning-induced neuroprotection is related to the suppression of the inflammatory response in the ischemic area of the brain, but the mechanisms involved in LPS preconditioning are poorly understood [12,13].

MCPIP1 (also known as ZC3H12A) is a recently identified protein in human peripheral blood monocytes treated with monocyte chemotactic protein 1 (MCP-1) [14]. In our previous studies, MCPIP1 was shown to be a negative regulator of macrophage activation [15]. Further investigations by our group and others indicated that MCPIP1 can play a significant anti-inflammatory role by inhibiting the generation of a set of major proinflammatory cytokines [16,17]. MCPIP1 was also found to be inducibly expressed in monocytes, macrophages, and endothelial cells with LPS stimulation [13,17-19]. However, the role of MCPIP1 in ischemic stroke has not been examined. In this study we examined MCPIP1 gene expression in human and mouse microglia, and in mouse brain under LPS treatment or preconditioning. We also examined MCPIP1 gene expression in mouse brain undergoing MCAO. We studied whether there is loss of LPS preconditioning-induced ischemic stroke tolerance in MCPIP1 knockout mice and whether such effects involve regulation of expression of proinflammatory cytokines. Furthermore we investigated the effects of MCPIP1 on JNK signal pathway under brain ischemia conditions and the effects of JNK inhibitor on the production of proinflammatory cytokines in MCPIP1 knockout mice after brain ischemia. Our data indicate that MCPIP1 is upregulated under LPS preconditioning or after brain ischemia stress and MCPIP1 participates in LPS preconditioning-induced ischemic stroke tolerance by modulating gene expression of proinflammatory cytokines.

## Methods

### Animals and LPS preconditioning

MCPIP1 knockout mice were established as previously described [16]. Briefly, Mcpip1^-/- ^mice was generated by homologous recombination in embryonic stem cells from C57/BL6 background mice. Exons 3, 4, 5 and most part of 6 of mouse Mcpip1 were targeted with a LacZ-neomycin cassette in embryonic stem cells established from C57/BL6 mice and established Mcpip1^-/- ^mice in pure C57/BL6 background. The deletion of MCPIP1 protein in Mcpip^-/- ^mice was confirmed by Immunoblotting. Six to eight-week-old mice were used. All experimental procedures were approved by the Institutional Animal Care and Use Committee of University of Central Florida. We performed all the experiments by using littermate mice. For in vivo study mice were given an intraperitoneal injection of saline or LPS (Sigma, USA) 0.2 mg/kg in saline 24 hours before transient middle cerebral artery occlusion (MCAO) [12].

### Cell cultures

Human microglia was acquired from Clonexpress, Inc. (Gaithersburg, MD, USA) and cultured in 50:50 DMEM: F-12 supplemented with 10% FBS and 10 ng/ml of M-CSF, grown at 37°C in a humidified environment (5% CO_2_; 95% air). Mouse microglia cultures were generated by the procedures described by Watson and colleagues [20]. In brief, mouse microglia was prepared from cortices of C57BL/6 mouse brain. Brain tissue was placed in 2 ml Dulbecco's modified Eagle's medium (DMEM; Invitrogen, USA) supplemented with 10% fetal bovine serum (Gibco, USA), penicillin (100 U/ml; Gibco, USA) and streptomycin (100 U/ml; Gibco, USA). Samples were triturated, passed through a sterile nylon mesh filter; centrifuged (1500 rpm, 5 min, 20-22°C) and the pellets were resuspended in DMEM. Cells were grown in T25 flasks in DMEM medium supplemented with 10% FBS. After 12 days the flasks were shaken for 2 hours at 110 rpm at room temperature and tapped several times to remove the non-adherent microglia. The supernatant was centrifuged at 1500 rpm for 5 min and the pellet was resuspended in normal DMEM medium mentioned above for experiments. LPS (Catalog #, L4516, Sigma, USA) or PBS was added to medium (0.1 μg/ml) for cell stimulus experiments.

### Mouse focal brain ischemia reperfusion model

For focal brain ischemia, mouse reversible middle cerebral artery occlusion (MCAO) was produced by filament occlusion of the right MCA following a modification of the method reported by Clark and colleagues [21]. In brief, mice were anesthetized with isoflurane (induction with 3%; maintenance with 1.2%) in oxygen-enriched air by facemask, and rectal temperature was controlled at 37 ± 0.5°C throughout the experiment with heating lamps. Unilateral MCAO was performed by inserting a 7-0 nylon monofilament into the internal carotid artery via an external carotid artery stump and then positioning the filament tip for occlusion at a distance of 8-9 mm beyond the internal carotid/pterygopalatine artery bifurcation. MCA was occluded for 90 minutes followed by reperfusion.

### Brain infarction measurement

The brains were stained with 2,3,5-triphenyltetrazolium chloride (TTC) (Sigma, USA) to determine infarct volume [4,22]. After 90 min of MCAO and 48 hours of reperfusion, mice were anesthetized with 4% isoflurane and brains were removed and sectioned coronally at a thickness of 2 mm and incubated in 2% TTC at 37°C for 20 minutes. Brain slices were then fixed in 4% paraformaldehyde at 4°C overnight and scanned into a computer, and quantified using the Image J software. Infarct volume was expressed as a percentage of the contralateral hemisphere. There were 10 mice in each group.

### Brain edema measurement

The mice were anesthetized with 4% isoflurane and brains were removed at different time points, i.e., 12, 24, and 48 h after MCAO. The brains were weighed to obtain the wet weight and were then dried at 105°C for 24 h before measuring dry weight. Brain moisture content (%) was calculated as follows, 100 × (wet weight- dry weight)/wet weight. There were ten mice in each group.

### Quantitative real-time PCR

Quantitative Real Time-PCR was performed as previously described [15]. Briefly, Total RNA was isolated using RNA STAT-60 reagent (TEL-TEST, INC. USA), after removing the genomic DNA using DNase I (Ambion, USA), 2.0 ug of total RNA from microglia or mouse brain tissue was reverse-transcribed to cDNA using a commercially available kit (Applied Biosystems, USA). Quantitative real-time PCR was performed with iCycler Thermal Cycler (Bio-Rad, USA) using 2 × SYBR Green master mixes (Bio-Rad, USA). Forty cycles were conducted as follows: 95°C for 30 s, 60°C for 30 s, proceeded by 10 min at 95°C for polymerase activation. Quantification was performed by the delta cycle time method, with mouse β-actin used for normalization. Human MCPIP1 gene specific primers (IDT, USA) were F: 5'-GCCGGCGGCCTTA; R: 5'-GCACTGCTCACTCTCTGTTAGCA. The mouse specific primers (IDT, USA) are as follows, MCPIP1: F: 5'-CCCCCTGACGACCCTTTAG; R: 5'- GGCAGTGGTTTCTTACGAAGGA, TNFα: F: 5'- CTGAGGTCAATCTGCCCAAGTAC; R: 5'-CTTCACAGAGCAATGACTCCAAAG, IL-1β: F: 5'- GCCCATCCTCTGTGACTCAT; R: 5'- AGGCCACAGGTATTTTGTCG, IL-6: F: 5'- TCGTGGAAATGAGAAAAGAGTTG; R: 5'- AGTGCATCATCGTTGTTCATACA, MCP-1: F: 5'- CCATCTCTGACCTGCTCTTCCT; R: -AGACCCACTCATTTGCAGCAT, β-actin: F: 5'- AAATCGTGCGTGACATCAAAGA; R: 5'- GGCCATCTCCTGCTCGAA.

### Western blot

Western blot was performed as previously described [15]. Proteins from microglia or mouse brain tissue were extracted and concentrations were determined by the Bradford method (Bio-Rad, USA) with bovine serum albumin as the standard. Proteins (50 ug) were separated by SDS-PAGE and transferred onto nitrocellulose membranes in transfer buffer containing 0.1% SDS. The membranes were blocked with 5% nonfat dry milk in 0.05% Tween 20 in Tris-buffered saline (TTBS) for 2 h and incubated with the primary antibodies against MCPIP1 (Catalog #, sc136750, Santa Cruz, USA), phosphor-SAPK/JNK (Catalog #, 9251, Cell Signaling, USA), SAPK/JNK (Catalog #, 9252, Cell Signaling, USA), phosphor-c-jun (Catalog #, 2361, Cell Signaling, USA), c-jun (Catalog #, 2315, Cell Signaling, USA) at a 1:1000 dilution in the blocking buffer, 4°C, gently shaking, overnight. After being washed with TTBS three times for 10 min each, the membranes were incubated with a 1:2,000 dilution of secondary antibody (Santa Cruz, USA) in TTBS for 1 h. Following three 10-min washes with TTBS, membranes were incubated with SuperSignal West Pico Chemiluminescent Substrate (Pierce, USA) and exposed to x-ray film. The intensity of bands was quantified by AlphaImage 2200 (AlphaInnotech, USA). The ratios between interested protein bands and the loading control (β-actin, total JNK or c-jun) were calculated and the data are expressed as the normalized folds with respect to sham.

### Drug administration

JNK specific inhibitor SP600125 (Sigma, USA) was dissolved in PPCES vehicle (30% polyethylene glycol-400/20% polypropylene glycol/15% cremophor EL/5% ethanol/30% saline) as reported [23] and was treated by mice tail-vein injection 1 h before ischemia at a dose of 15 mg/kg.

### Statistical analysis

The data are presented as mean ± SD. Multiple comparisons were evaluated by one-way ANOVA followed by the Tukey or Dunnett test. Two-group comparisons were analyzed by the 2-tailed Student t test. For all analyses, a value of P < 0.05 was considered significant.

## Results

### MCPIP1 induction in mouse and human microglia by LPS treatment

**Figure 1:**
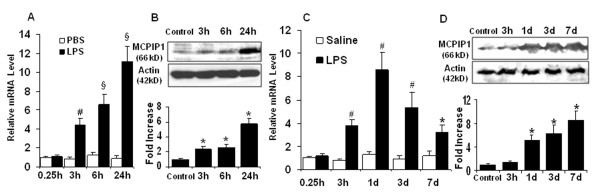
**MCPIP1 mRNA and protein levels are elevated in mouse microglia and brain upon treatment with LPS**. (**A**) MCPIP1 mRNA expression in mouse microglia treated with LPS (0.1 μg/ml) as measured by qRT-PCR. Values represent mean ± SD, # *p *< 0.01, § *p *< 0.001 versus PBS control. (**B**) MCPIP1 protein levels in mouse microglia treated with LPS (0.1 μg/ml) as measured by Western blot. Results are representative of three independent experiments. *p < 0.05 versus PBS control. (**C**) MCPIP1 mRNA expression in mouse brain with LPS-preconditioning (0.2 mg/kg) as measured by qRT-PCR. Values represent mean ± SD, # *p *< 0.01, § *p *< 0.001 versus saline control. (**D**) MCPIP1 protein levels in mouse brain with LPS-preconditioning (0.2 mg/kg) as measured by Western blot. Results are representative of three independent experiments. *p < 0.05 versus saline control.

**Figure 2:**
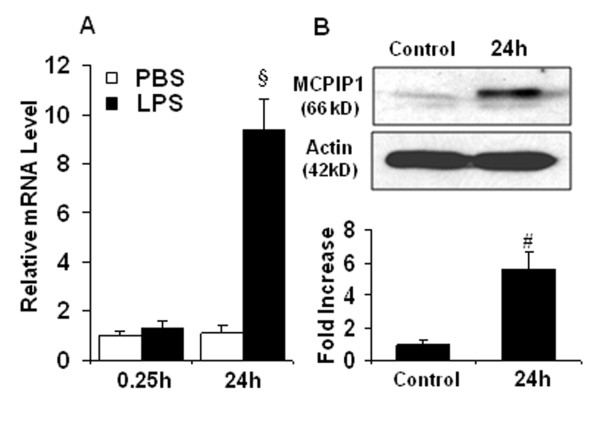
**MCPIP1 mRNA and protein increase in human microglia treated with LPS**. (**A**) MCPIP1 mRNA expression in human microglia treated with LPS (0.1 μg/ml) as measured by qRT-PCR. Values represent mean ± SD, § *p *< 0.001 versus PBS control. (**B**) MCPIP1 protein levels in human microglia treated with LPS (0.1 μg/ml) as measured by Western blot. Results are representative of three independent experiments. # *p *< 0.01 versus PBS control.

In ischemic stroke, microglia is activated after brain ischemia and release inflammatory cytokines that exacerbate brain injury. We examined whether LPS induces MCPIP1 in mouse microglia. The MCPIP1 mRNA level in mouse microglia was significantly induced by LPS (0.1 μg/ml) treatment compared to PBS controls; significant increase of MCPIP1 in transcript level was detected at 3 h and reached 11.12 ± 1.63 fold at 24 h after LPS treatment (*P *< 0.001; Figure 1A). Consistently, the MCPIP1 protein levels in mouse microglia were significantly higher in LPS (0.1 μg/ml) treated cells than in the controls, 5.93 ± 0.72 fold on 24 h after LPS treatment (P < 0.05; Figure 1B). The MCPIP1 mRNA level in human microglia was significantly higher in the LPS (0.1 μg/ml) group than that in the controls at 24 h after LPS treatment (9.35 ± 1.76 folds, *P *< 0.001; Figure 2A). The MCPIP1 protein level in human microglia was also significantly higher in LPS (0.1 μg/ml) group than that of control (5.65 ± 1.23 folds, *P *< 0.01; Figure 2B).

### MCPIP1 induction in mouse brain cortex after LPS preconditioning

To determine whether MCPIP1 expression is induced by LPS preconditioning in vivo, mice were treated systemically with 0.2 mg/kg LPS in saline and control mice with only saline as reported [12]. MCPIP1 mRNA levels significantly increased in the brain cortex as a result of LPS treatment. After 3 hr of LPS treatment the transcript levels showed substantial increase (3.81 ± 0.53 folds) and the maximal level was reached 24 hr (8.62 ± 1.51 folds) after LPS administration with subsequent decrease but remaining at 3.27 ± 0.59 folds level even after 7 days (Figure 1C). MCPIP1 protein levels significantly increased in the 24-hours after LPS treatment (5.23 ± 0.82 folds) and showed further increase until 7 days (Figure 1D). These results suggest that MCPIP1 is significantly elevated in the mouse brain by LPS preconditioning treatments.

### MCPIP1 induction in mouse brain cortex after MCAO

**Figure 3:**
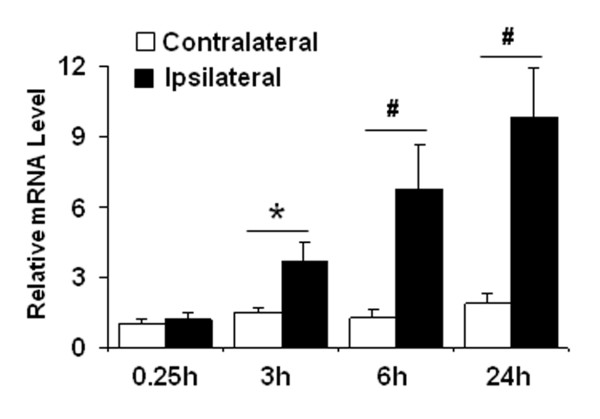
**MCPIP1 mRNA increases in ipsilateral brain after MCAO**. MCPIP1 mRNA expression in mouse brain was determined at 3 h, 6 h and 24 h after MCAO by qRT-PCR. Values represent mean ± SD, * *p *< 0.05, # *p *< 0.01 versus contralateral control. n = 5 per group.

We determined whether MCPIP1 is induced by brain ischemia stress. We examined MCPIP1 mRNA level in mouse brain cortex after MCAO. The MCPIP1 mRNA level in the ipsilateral side of mouse brain was significantly induced after MCAO compared to the contralateral side; significant increase of MCPIP1 in transcript level was detected at 3 h and reached 9.85 ± 2.1 fold at 24 h after MCAO (*P *< 0.01; Figure 3), suggesting that MCPIP1 may play an important role after brain ischemia stress.

### Loss of LPS-induced tolerance to ischemic stroke by MCPIP1 deficiency

**Figure 4:**
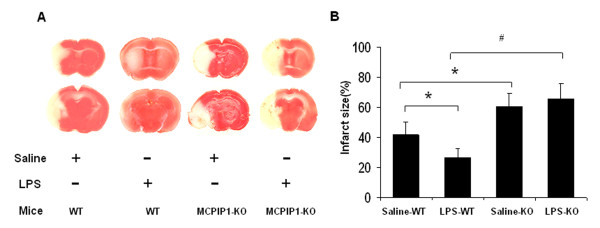
**Loss of LPS-induced brain ischemia tolerance in MCPIP1 KO mice**. Wild type and MCPIP1 knockout mice were pretreated with LPS (0.2 mg/kg) 24 hours before MCAO. **(A) **Infarct images obtained by TTC staining at 48 h after MCAO. The normal tissue was stained deep red and the infarct was stained milky. **(B) **Brain infarcts were assessed 48 hours after MCAO and quantified as percentage area of ischemic hemisphere. Values represent mean ± SD, *p < 0.05, # *p *< 0.01 versus wild type, n = 8 mice per group.

We examined the effects of low dose (0.2 mg/kg) of LPS on ischemic brain infarction and the results showed that the infarct size of LPS-preconditioned mice was significantly reduced compared to that of saline control. (41.5 ± 10.1% versus 26.3 ± 6.4%, Figure 4A, B). To determine whether MCPIP1 is involved in LPS preconditioning-induced tolerance to ischemic brain injury, MCPIP1 knock out or wild type mice were treated with a low dose of LPS (0.2 mg/kg), and 24 hours later these mice were subjected with MCAO for 90 min followed by 48 hours reperfusion. The brain infarct size was assessed with TTC staining 48 hours after MCAO. MCPIP1 knockout mice failed to evoke LPS-induced tolerance compared with that of wild type (65.6 ± 12.1% versus 26.3 ± 6.4%, Figure 4A, B). There was no significant difference in brain infarct between LPS-preconditioning and control in MCPIP1 knockout mice.

### Brain edema and mortality associated with ischemia/reperfusion (I/R) injury are exacerbated by absence of MCPIP1

**Figure 5:**
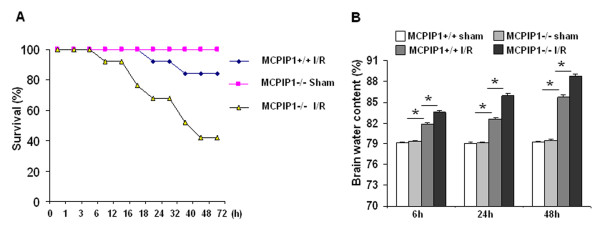
**MCPIP1 knockout mice in higher mortality and exacerbated edema to brain ischemia/reperfusion caused injury. (A) **Eight-week-old MCPIP1 KO and littermate WT mice were subjected to MCAO for 90 minutes followed by 72 hours of reperfusion; sham-operated mice had the same procedures except no MCA occlusion. Survival was monitored for 72 hours after the MCAO. MCPIP1 knock out I/R mice showed lower survival than wild type I/R (*p *< 0.05) and sham-operated MCPIP1 knock out mice (*p <*0.05). n = 12 mice per group. (**B**) Brain water content as a measure of brain edema of the ischemic hemisphere. MCPIP1 knockout mice had a significant increase in brain edema at 6, 24, and 48 h after MCAO; Values represent mean ± SD,* *p *< 0.05 versus wild type group. n = 10 per group.

To investigate the possible role of MCPIP in ischemic injury in the brain we subjected MCPIP1 knockout mice to reversible middle cerebral artery occlusion (MCAO) for 90 minutes followed by reperfusion for 72 hours. Edema is one of the earliest pathological changes after ischemic neuronal damage, which significantly increases as early as 20 to 45 mins after MCAO [24]. Our results show that brain edema was enhanced in a time-dependent manner after MCAO in wild type mice (6 h: 81.31 ± 0.19%; 24 h: 82.78 ± 0.23%; 48 h: 85.34 ± 0.33%, Figure 5B) as compared with the sham-treated group (48 h: 79.47 ± 0.21%). However, MCPIP1 knockout mice showed a significantly higher increase in brain edema after MCAO (6 h: 83.52 ± 0.27%; 24 h: 85.73 ± 0.31%; 48 h: 88.69 ± 0.38%, Figure 5B). MCPIP1 knockout mice showed significantly lower survival than the wild type and sham-operated MCPIP1 knockout mice (p < 0.05, Figure 5A).

### Increased proinflammatory cytokine expression

**Figure 6:**
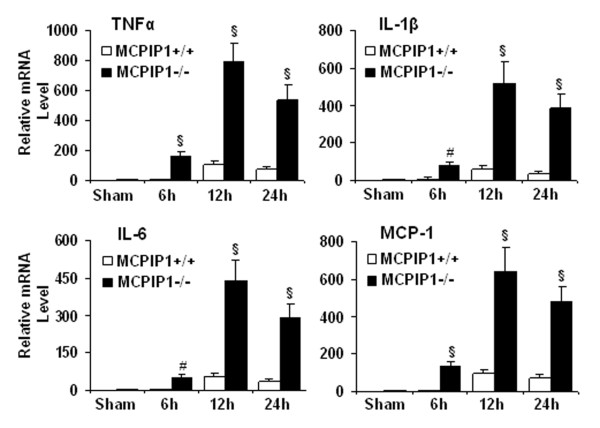
**Proinflammatory cytokine expression in the ischemic brain**. MCPIP1deficiency significantly elevated the expression of proinflammatory cytokines TNFα, IL-1β, IL-6 and MCP-1, as measured by q-RT-PCR in the ischemic brain after MCAO. Values represent mean ± SD, # *p *< 0.01 and § *p *< 0.001 versus wild type, n = 6 mice per group.

We examined the expression of proinflammatory cytokine transcript in the ischemic brain of the wild type and MCPIP1 knockout mice after MCAO. TNFα, IL-1β, IL-6 and MCP-1 expression was substantially upregulated after brain ischemia. Different cytokines showed similar temporal pattern of this upregulation. These cytokines were more highly induced in MCPIP1 knockout mice compared with that of wild type 6 h, 12 h and 24 h after MCAO (Figure 6).

### Activation of c-jun N terminal kinase (JNK) signaling pathway

**Figure 7:**
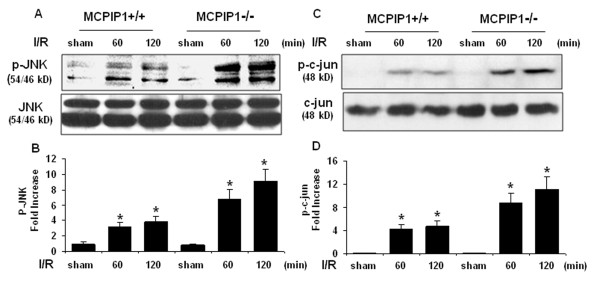
**Activation of c-Jun N terminal kinase (JNK) signaling pathway**. Proteins extracted from the ischemic hemisphere of wild type and MCPIP1 knockout mice undergoing ischemia 30 min followed by 30 and 90 min reperfusion separately. The samples were subjected to Western blot analysis with antibodies as indicated. (**A**) A representative western blot shows protein levels of JNK phosphorylation. **(B) **Densitometric analysis was used to quantify p-JNK protein levels versus total JNK in 3 independent western blots and the data are expressed as the normalized folds with respect to sham. Values represent mean ± SD, *p < 0.05 versus sham-treated control. **(C) **A representative western blot shows protein levels of c-jun phosphorylation. **(D) **Densitometric analysis was used to quantify p-c-cun protein levels versus total c-jun in 3 independent western blots and the data are expressed as the normalized folds with respect to sham. Values represent mean ± SD, *p < 0.05 versus sham-treated control.

JNK/c-jun signaling mediates a wide spectrum of cellular responses, including infection, inflammation, and apoptosis [25]. To understand the mechanisms underlying the increased inflammatory response of MCPIP1 knockout mice after brain ischemia, we examined the activation of JNK/c-jun signaling pathway. The phosphorylation of JNK and c-jun significantly increased in mice after brain ischemia/reperfusion and absence of MCPIP1 caused marked elevation in the levels of both JNK and c-jun phosphorylation after ischemia/reperfusion compared with that of wild type (Figure 7A, B, C, D).

### Knocking out MCPIP1 increases proinflammatory cytokines via JNK signaling cascade

**Figure 8:**
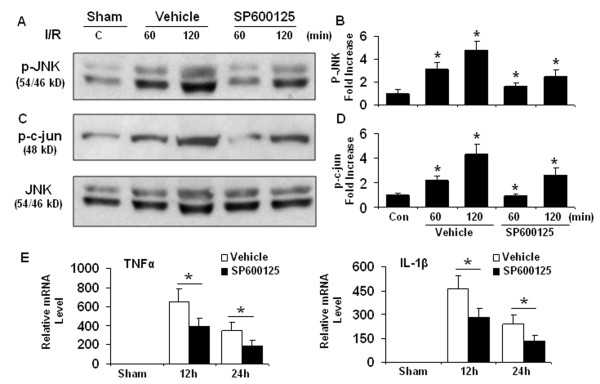
**JNK signaling plays a critical role in proinflammatory cytokines production**. MCPIP1 knockout mice were treated with SP600125 (15 mg/kg, iv) 60 min prior to MCAO and proteins extracted from the ischemic hemisphere of MCPIP1 knockout mice undergoing ischemia 30 min followed by 30 and 90 min reperfusion separately. (**A**) A representative western blot shows protein levels of JNK phosphorylation. **(B) **Densitometric analysis was used to quantify p-JNK protein levels versus total JNK in 3 independent western blots and the data are expressed as the normalized folds with respect to sham. Values represent mean ± SD, *p < 0.05 versus sham-treated control. **(C) **A representative western blot shows protein levels of c-jun phosphorylation. **(D) **Densitometric analysis was used to quantify p-c-cun protein levels versus total JNK as the loading control in 3 independent western blots and the data are expressed as the normalized folds with respect to sham. Values represent mean ± SD, *p < 0.05 versus sham-treated control. **(E) **The expression of proinflammatory cytokines was determined at 12 h and 24 h after MCAO and inhibition of JNK activation significantly reduced mRNA levels of TNFα and IL-1β in the ischemic hemisphere of MCPIP1 knockout mice undergoing MCAO. Values represent mean ± SD,* *p *< 0.05 versus sham group. n = 5 per group.

We determined whether the increased proinflammatory cytokine production is mediated via JNK and c-jun signaling pathway. We performed specific JNK inhibition experiments in vivo with JNK inhibitor SP600125 in MCPIP1 knock out mice after MCAO. MCPIP1 knock out mice received the JNK inhibitor SP600125 (15 mg/kg) before 60 min of transient focal ischemia. SP600125 treatment caused a significant decrease in the levels of both JNK and c-jun phosphorylation (Figure 8A, B, C, D) and the expression of proinflammatory cytokines measured at 12 h and 24 h after MCAO compared with vehicle group (Figure 8E). These results support the hypothesis that activation of JNK signaling may be an integral component of the mechanism underlying the excessive generation of proinflammatory cytokines after ischemic brain injury.

## Discussion

It is becoming increasingly clear that inflammation and innate immune response play an important role in the brain injury after ischemic stroke [26,27]. Inflammatory mechanisms that are activated within hours after brain ischemia represent a key target for stroke intervention. Although it has been known that LPS preconditioning can induce significant tolerance to ischemic brain injury and inhibit inflammatory responses such as activation of microglia, brain neutrophil infiltration and proinflammatory cytokine generation, the molecular mechanisms that contribute to brain ischemia tolerance by LPS preconditioning are not well-understood. The present study is the first to examine the role of MCPIP1 in acute cerebral ischemia. We found that MCPIP1 can be significantly induced in human and mouse microglia and mouse brain with LPS stimulus or preconditioning and that MCPIP1 gene expression significantly increased in mouse brain after MCAO. These findings are consistent with the significance of inflammatory processes contributing to the brain damage in ischemic stroke [26] and the anti-inflammatory properties of MCPIP1 [16,17]. Similarly we also found the upregulation of MCPIP1 in cultured mice astrocytes following LPS stimulation (data not shown) and activated astrocytes also play an important role in neuroinflammation [28]. Thus, we conclude that MCPIP1 is involved in LPS preconditioning-induced ischemic stroke tolerance by its anti-inflammatory activities and MCPIP1 may play an important role against ischemic brain injury. Consistent with other reports [7,12] we found that a small dose of LPS given systemically confers protection against ischemia in mouse brain with respect to infarct volume. More interestingly, we found that there was loss of LPS-induced ischemic stroke tolerance by MCPIP1 deficiency. There was much larger ischemic brain infarct area in MCPIP1 deficient mice compared to the wild type. Higher mortality in MCPIP1 knockout mice subjected to focal brain ischemia/reperfusion injury compared with wild type indicated that MCPIP1 deficient mice is more sensitive to ischemic brain injury than wild type. This result was not due to the possible general physiological weakness of MCPIP1 knockout mice to resist stress of surgery as mortality of sham group of MCPIP1 knockout mice was much less than the animals subjected to ischemic injury.

Clinical data showed that the principal cause of stroke death in patients with malignant middle cerebral artery infarction was focal brain edema [29]. Experimental models of cerebral infarction show an increase in water content beginning within hours and peaking at 48 hours [30]. Progressive postischemic edema would result in increased intracranial pressure with compression of the brain stem, occlusive hydrocephalus and secondary ischemic damage finally followed by clinical deterioration, coma, and death within 2 to 5 days after stroke [31,32]. In this study, our data showed that absence of MCPIP1 significantly increased the infarct volume and brain edema compared to wild type after ischemia/reperfusion injury. Thus, severe brain edema may be the major cause of higher mortality in MCPIP1 knockout mice subjected to ischemic stroke.

Inflammatory response after stroke significantly contributes to ischemic brain damage, which can be sub-divided into the cellular response and the cytokine response [6]. Neutrophils accumulate in the brain as early as 30 min after permanent middle cerebral artery occlusion (MCAO). Transmigration is mediated by cell adhesion molecules such as vascular cell adhesion molecule (VCAM)-1, intercellular adhesion molecule (ICAM)-1, and selectin, which contribute to the recruitment of inflammatory cells to endothelial blood vessel wall [33,34]. When adhered to cerebral blood vessel walls, neutrophils transmigrate into the cerebral parenchyma, which can cause tissue damage by releasing reactive oxygen species and proteolytic enzymes. MCPIP1 expression is induced in human blood endothelial cells and overexpression of MCPIP1 suppresses cytokine-induced expression of VCAM-1, as well as monocyte adhesion to human endothelial cells [19]. Such properties of MCPIP1 helps to explain the present results that absence of MCPIP1 exacerbates ischemia induced brain injury.

Proinflammatory cytokines and chemokines contribute to stroke-related brain injury [6,35]. During ischemia, cytokines, such as TNF-α, IL-1β, IL-6, and chemokines such as CINC and MCP-1 are produced by a variety of activated cell types, including endothelial cells, microglia, astrocytes and neurons [36]. The deleterious effects of these cytokines include fever, arachidonic acid release, enhancement of NMDA mediated excitotoxicity, and stimulation of nitric oxide synthesis. TNFα and IL-1 have been shown to cause up-regulation of E-selectin, ICAM-1, ICAM-2, and VCAM-1 on cerebral endothelial cells and the induction of such adhesion molecules may explain the elevation of TNFα and IL-1 levels after ischemia increases neutrophil infiltration [37]. Additionally TNFα can stimulate acute-phase protein production, including plasminogen, disrupt the blood-brain barrier and stimulate the induction of other inflammatory mediators. LPS preconditioning can induce significant tolerance to ischemic brain injury and inhibit inflammatory responses such as activation of microglia, brain neutrophil infiltration and proinflammatory cytokine generation. Some studies attributed LPS preconditioning to reprograming of cellular response to ischemia via genomic changes that render the brain refractory to ischemic injury [11,38]. However, the molecular mechanisms that mediate the beneficial effects in LPS-induced ischemic tolerance remains poorly understood. In this study we observed that knocking out MCPIP1 increased the brain damage after ischemic stroke and significantly enhanced the expression of proinflammatory cytokines in the brain, which may account for the severe brain damage resulting from ischemia in MCPIP1 deficient mice. It has been established that endotoxin-preconditioning by LPS, TNFα and IL-1β can induce ischemic tolerance. Less well studied is what mediates such beneficial tolerance in preconditioning process i.e. the necessary intermediate links for completion of preconditioning tolerance. If this link is lost, the chain from cytokines to tolerance would be broken; in other words, even if cytokines still exist the tolerance would be not induced or would be inhibited. We hypothesized that MCPIP1 is such an inducible factor or link which mediates, at least in part, the translation from cytokine stimulation to tolerance. When MCPIP1 is deficient, tolerance is reduced even in the presence of more cytokines, as the key mediator is missing. Our results suggest that MCPIP1 is such a mediating factor and MCPIP1-deficiency results in the loss of LPS-induced ischemia tolerance in the brain and higher level of proinflammatory cytokines in ischemic brain. How does MCPIP1 regulate these inflammatory cytokine responses remains to be fully elucidated. It has been reported that MCPIP1 might be functioning as an RNase to promote the degradation of some inflammatory mRNA such as IL-6 and IL-1β [17]. We have found that MCPIP1 can also act as a deubiquitinase to negatively regulate JNK and NF-κB signaling by targeting TNF receptor-associated factors (TRAFs) [16,39], which suggests that MCPIP1 may control inflammatory response by multiple mechanisms. Activation of JNK signaling pathways leads to c-Jun mediated inflammatory cytokine production [24,40,41]. In this study we found that knocking out MCPIP1 significantly enhanced the activation of JNK kinase and phosphorylation of c-jun in mouse brain after ischemic/reperfusion injury and that administration of the JNK inhibitor SP600125 reduced hyperphosphorylation of JNK and c-Jun signaling after cerebral ischemia and significantly decreased the production of proinflammatory cytokines such as TNFα and IL-1β. Our study suggests that increased activation of JNK signaling pathway in MCPIP1 knockout mice leads to increased proinflammatory cytokine production.

## Conclusions

Based on the data, we concluded that MCPIP1 participates in LPS preconditioning-induced ischemic stroke tolerance. Knocking out MCPIP1 gene exacerbates brain damage after cerebral ischemia/reperfusion by upregulation of proinflammatory cytokines.

## Competing interests

The authors declare that they have no competing interests.

## Authors' contributions

JL designed the experiments, performed all experiments, analyzed the data, generated the figures, and wrote the manuscript. JW did parts of the animal surgery. JW, YS, LW, EB performed the experiments. PEK provided advice in the design of the study and in interpreting of data and critically read and corrected the manuscript. All authors have read and approved the manuscript.
